# A unified fixed-wavelet transformer decodes eight EEG classes across motor imagery, P300, and SSVEP paradigms

**DOI:** 10.3389/fnhum.2026.1973838

**Published:** 2026-10-29

**Authors:** Woo-Sung Choi, Hong-Gi Yeom

**Affiliations:** 1 Department of Electronics Engineering, Chosun University, Gwangju, Republic of Korea; 2 Interdisciplinary Program in IT-Bio Convergence System, Chosun University, Gwangju, Republic of Korea

**Keywords:** brain–computer interface, cross-paradigm EEG decoding, electroencephalography, explainable AI, heterogeneous paradigm learning

## Abstract

**Introduction:**

Motor imagery (MI) can support self-initiated control, whereas brain–computer interfaces (BCIs) based on the P300 or the steady-state visual evoked potential (SSVEP) rely on ongoing visual stimulation. Repetitive visual stimulation can cause visual fatigue; when the stimuli are suspended, however, the corresponding P300 and SSVEP responses can no longer be elicited, and these paradigms alone cannot support a self-initiated command to restart visual stimulation. By contrast, self-initiated MI can provide a voluntary gating signal for switching visual stimulation on or off. Developing a single architecture capable of learning these heterogeneous EEG signatures and decoding multiple user intentions is therefore an important step toward a voluntarily controlled hybrid BCI.

**Methods:**

To this end, we developed a unified model by extending our previously proposed Analformer, a Transformer-based architecture designed for high classification performance while enabling the analysis of neurophysiologically meaningful EEG representations through AnalNet. We evaluated whether the model could classify EEG trials into eight mutually exclusive classes drawn from MI, P300, and SSVEP paradigms in the OpenBMI dataset. Analformer uses fixed Morlet wavelet kernels to extract frequency-specific EEG features, six Transformer blocks model dependencies across 31 pooled temporal tokens, and a two-stage training procedure to capture both population-level and participant-specific features.

**Results:**

Across 54 participants, the mean accuracy obtained from the model after participant-specific fine-tuning was 71.00%, compared with 54.53% for EEG-Conformer, 52.30% for EEGNet, and 45.35% for ShallowConvNet under the same evaluation protocol. Ablation analyses showed that replacing the fixed wavelets with learnable convolutions reduced the mean accuracy to 63.15%, whereas omitting the two-stage training procedure reduced it to 47.38%. AnalNet, the built-in analysis module of Analformer, identified class-discriminative patterns, including sensorimotor mu- and beta-band activity during MI and stimulus-locked occipital responses during SSVEP.

**Discussion:**

Together, these findings indicate that a single Transformer-based architecture can distinguish heterogeneous EEG classes and provide a foundation for unified decoding in hybrid BCIs that combine self-initiated MI gating with P300- and SSVEP-based command selection.

## 1 Introduction

Brain–computer interfaces (BCIs) translate neural activity into commands for external devices or computer systems (Yeom et al., 2013; Choi and Yeom, 2022; Wang et al., 2026). By bypassing conventional muscular output, BCIs can restore communication or motor control for people with paralysis and provide a direct channel for human–machine interaction.

Electroencephalography (EEG) is widely used for non-invasive BCI because it combines millisecond temporal resolution with relatively low acquisition cost (Rashid et al., 2020; Liu et al., 2025; Elwasify et al., 2026). Three established EEG paradigms encode intention through different neural signatures and interaction modes. Motor imagery (MI) modulates sensorimotor rhythms (SMR) (Pfurtscheller and Da Silva, 1999; Scherer and Vidaurre, 2018); event-related potentials (ERP), including the P300, appear as stimulus-locked amplitude deflections (Polich, 2007; Fazel-Rezai et al., 2012; Haider and Fazel-Rezai, 2017); and steady-state visually evoked potentials (SSVEP) produce frequency-locked responses to periodic visual stimulation (Vialatte et al., 2010; Norcia et al., 2015).

These interaction modes impose complementary constraints on practical BCI control. P300 and SSVEP interfaces can support many selectable symbols or directions, but they depend on flashing or flickering visual stimuli. ERP and SSVEP tasks produce higher eye-fatigue ratings than MI (Lee et al., 2019), whereas suspending the stimuli removes the evoked responses on which these reactive interfaces depend and therefore prevents the visual paradigm alone from providing a self-initiated command to reactivate itself. By contrast, SMR-based MI can be initiated without continuous external stimulation and can provide a self-paced control signal, although it commonly supports fewer reliably separable commands. A practical hybrid BCI could therefore use an SMR intention to activate or deactivate a command-rich P300 or SSVEP interface, reducing unnecessary visual stimulation while preserving on-demand access (Pfurtscheller et al., 2010; Nicolas-Alonso and Gomez-Gil, 2012).

Deep learning can extract task-relevant features directly from EEG, while Transformer architectures extend this approach by modeling long-range dependencies in biosignals (Anwar et al., 2025). Models ranging from shallow convolutional networks (Schirrmeister et al., 2017) and EEGNet (Lawhern et al., 2018) to EEG-Conformer (Song et al., 2022) and other attention- and Transformer-based architectures have performed strongly on paradigm-specific benchmarks (Ding et al., 2024; Liao and Wang, 2024; Jin et al., 2025; Wang and Wei, 2025; Zhang et al., 2025; Yeom et al., 2026).

However, reviews of EEG decoding show that most published models remain task- or paradigm-specific (Roy et al., 2019; Raza and Yusoff, 2025). Accordingly, the key research challenge is to determine whether a unified decoder can distinguish SMR, ERP, and SSVEP classes despite their distinct temporal, spectral, and spatial characteristics. Establishing this capability is an important step toward coordinating self-initiated mode control with command-rich stimulus-driven interaction in a unified BCI.

A concrete implementation is hierarchical mode control: an SMR-derived command activates a P300 speller or SSVEP direction panel, the stimulus-driven paradigm supplies the larger selection set, and a second SMR command suspends visual stimulation. This division assigns self-initiation to SMR and command-rich selection to P300 or SSVEP only during active use. By limiting flashing or flickering to user-requested periods, the hierarchy could reduce unnecessary visual exposure while preserving on-demand typing or movement control. A shared decoder for SMR, P300, and SSVEP is the classifier core required to test this control logic within one architecture.

Motivated by the complementary roles of self-initiated MI and stimulus-driven P300/SSVEP, this study investigates a unified prediction framework that distinguishes their heterogeneous EEG responses within a single architecture. The framework jointly models four SSVEP, two MI, and two ERP classes in a shared eight-class output space. We implement this unified cross-paradigm decoding approach using Analformer, our previously developed architecture for EEG decoding and neuroscientific analysis, which combines fixed Morlet wavelet representations for interpretability with Transformer-based learning (Yeom et al., 2026). AnalNet, Analformer’s built-in analysis module, further examines the class-discriminative patterns learned across the three paradigms.

To learn a cohort-level representation before individual adaptation, we used Session 1 in two stages: pooled pre-training across all 54 participants followed by separate adaptation to each participant’s Session 1 data. After participant-specific adaptation, the final model was evaluated once on that participant’s Session 2 data. This design directly tests whether one unrestricted output space can distinguish eight mutually exclusive MI, ERP, and SSVEP classes in the intended personalized cross-session setting. The result is the shared classifier core needed to coordinate SMR-based mode control with P300/SSVEP selection.

## 2 Related work

Conventional deep-learning BCI models generally infer user state within a single paradigm. EEGNet, DeepConvNet, and ShallowConvNet are representative convolutional architectures (Schirrmeister et al., 2017; Lawhern et al., 2018), whereas EEG-Conformer combines convolution and self-attention to learn task-specific EEG representations (Song et al., 2022).

Subsequent architectures have targeted more efficient feature extraction and longer temporal dependencies. Deep4Net learns hierarchical convolutional features (Schirrmeister et al., 2017), ShallowFBCSPNet approximates filter-bank common spatial pattern features, and temporal convolutional networks use dilated convolutions to extend the receptive field (Bai et al., 2018). EEG-ITNet combines inception-style temporal filters with interpretability-oriented analysis (Salami et al., 2022). ATCNet integrates attention mechanisms with temporal convolutions for EEG-based motor imagery classification (Altaheri et al., 2022).

These models have shown strong performance when applied to one EEG paradigm at a time, but they do not test a single classifier that must distinguish all eight classes drawn from MI, ERP, and SSVEP. The present study therefore evaluates whether such a classifier can learn the temporal, spectral, and spatial patterns needed to distinguish the labels across the three paradigms.

Prior work addresses different aspects of this problem. EEG-Inception uses cross-subject transfer learning for ERP decoding (Santamaria-Vazquez et al., 2020); S-JEPA learns transferable EEG features across datasets and evaluates them on downstream tasks spanning MI, ERP, and SSVEP (Guetschel et al., 2024); and SyncNet supports joint analysis of EEG/LFP datasets with different electrode layouts (Li et al., 2017). These studies demonstrate the value of transferable EEG representations across subjects, datasets, and electrode layouts, but none of them trained a single supervised classifier jointly on MI, ERP, and SSVEP labels.

From a feature-representation perspective, wavelet-based approaches have also been widely investigated for extracting informative time–frequency features from EEG signals. Recent work has explored adaptive and learnable wavelet formulations for motor imagery EEG decoding. Rational dilated wavelets have been applied to provide more flexible spectral representations (Siino et al., 2025), while RatioWaveNet introduced learnable wavelet parameters for adaptive time–frequency representation and interpretable spectral analysis (Siino et al., 2026).

Building on our earlier study, which introduced Analformer and evaluated the backbone separately on paradigm-specific decoding and neuroscientific-analysis tasks, the present contribution is the joint eight-class formulation and training protocol rather than a new backbone.

## 3 Methods

The cross-paradigm extension jointly trains on MI, ERP, and SSVEP trials, as summarized in Figure 1. After preprocessing and non-overlapping label encoding, each epoch enters an Analytical Patch Embedding module with fixed Morlet kernels. The evaluated temporal-attention configuration converts the pooled sequence into 31 tokens with 62 features per token, and a Transformer encoder models dependencies across these temporal positions before the classification head produces eight logits. AnalNet retains the first fixed-wavelet activations for time–frequency and *F*-value time–frequency (FTF) inspection (Yeom and Jeong, 2021).

**Figure 1:**
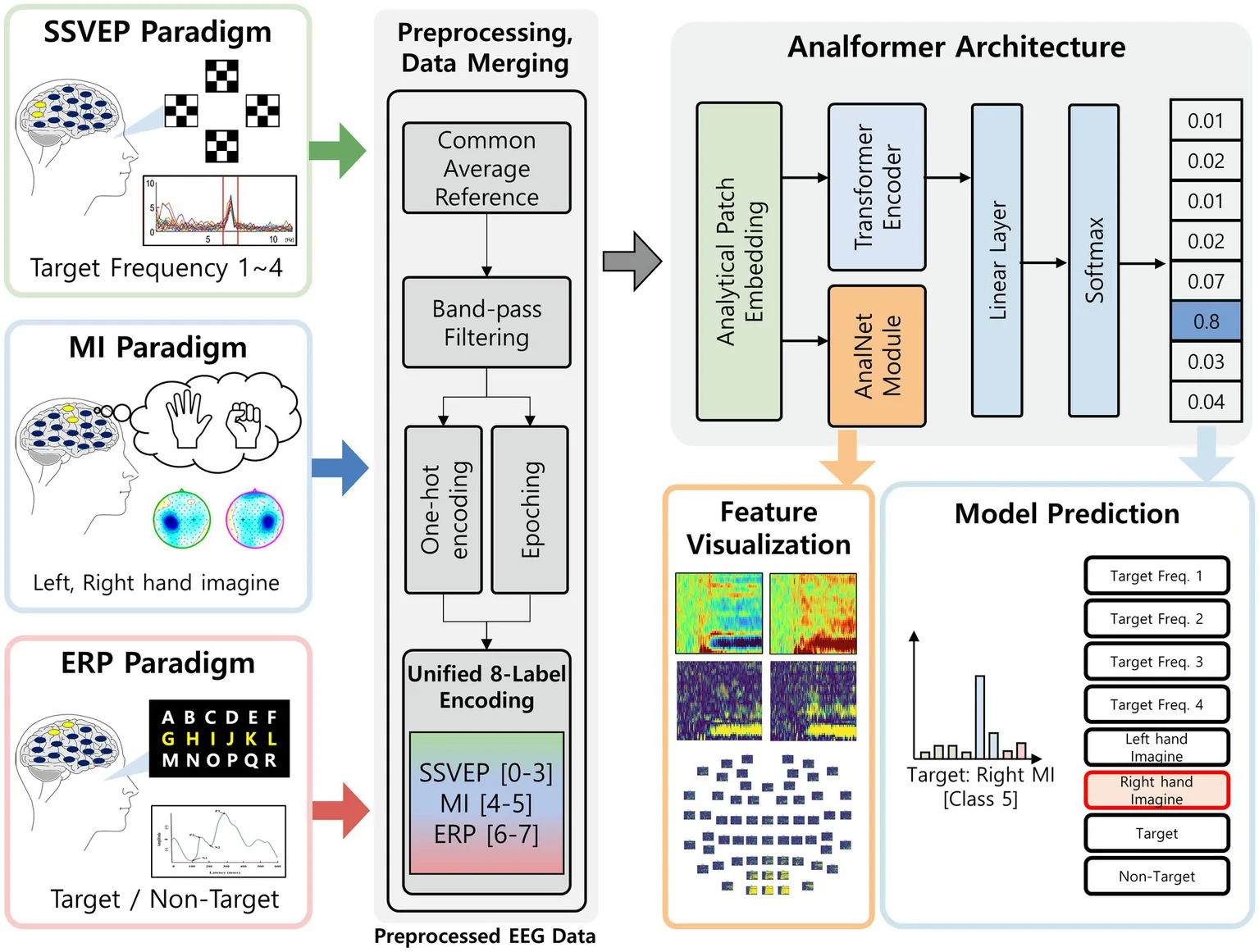
Overview of the multi-intention EEG decoding pipeline. Raw EEG signals from three paradigms (SSVEP, MI, and ERP) are preprocessed and integrated through a unified eight-label encoding scheme. Fixed Morlet kernels expose frequency-resolved features, learned convolutions produce a pooled sequence, and multi-head self-attention integrates 31 temporal tokens. The classification head produces eight logits, while AnalNet retains fixed-wavelet activations for time–frequency and FTF (*F*-value time–frequency) analysis.

### 3.1 Experimental dataset

All experiments used the publicly available OpenBMI dataset (Lee et al., 2019), which provides MI, ERP, and SSVEP recordings acquired from the same participants using the same EEG acquisition system. OpenBMI has been widely used as a benchmark dataset for evaluating BCI methods (Jin et al., 2025; Wang and Wei, 2025; Zhang et al., 2025; Yeom et al., 2026). This dataset comprises electroencephalography (EEG) recordings from 54 healthy adult participants (age 24–35 years; 25 females and 29 males), who completed all three BCI paradigms during each of two experimental visits. EEG signals were acquired using a 62-channel system at a sampling rate of 1,000 Hz, with electrode placement following the extended international 10–20 system.

In the SSVEP paradigm, participants attended to one of four visual stimuli flickering at 5.45, 6.67, 8.57, or 12 Hz for 4 s per trial. In the MI paradigm, participants imagined squeezing the left or right hand after a directional cue and performed the imagery task for 4 s after cue onset. In the ERP oddball paradigm, rare target images were interspersed among frequent non-target images using an 80 ms stimulus presentation and a 135 ms inter-stimulus interval. Both sessions were used: Session 1 from all participants supplied pooled pre-training data and subject-specific fine-tuning data, whereas Session 2 was reserved for final evaluation after adaptation.

### 3.2 Data preprocessing

A consistent preprocessing pipeline was applied to all paradigms and participants. Before training, the EEG data were common-average referenced, sixth-order Chebyshev Type I band-pass filtered from 0.5 to 40 Hz, and downsampled from 1,000 to 250 Hz. These settings retain low-frequency ERP activity, MI alpha/beta rhythms, and SSVEP responses while attenuating drift and higher-frequency activity.

For all paradigms, the input arrays contained 2 s epochs from −0.5 s to +1.5 s relative to stimulus onset, producing tensors of shape (62, 500); the −0.5 to 0 s interval is used as the prestimulus baseline for AnalNet normalization. ERP imbalance was controlled by retaining 100 target and 100 non-target epochs per participant and session. During pooled pre-training, the first 100 epochs of each ERP class were selected. For participant-specific adaptation and final evaluation, 100 epochs from each ERP class were randomly sampled without replacement from Session 1 and Session 2, respectively, for each participant, with the random seed used for each participant-level run recorded in the experiment log. The pre-training stage standardizes the pooled Session 1 tensor with one scalar mean and standard deviation. During personalization, the script computes one scalar mean and standard deviation from the participant’s merged Session 1 trials and applies those training-derived values to Session 2, so evaluation amplitudes do not determine the scaling. Each trial is stored as (1, 62, 500).

### 3.3 Unified eight-class label encoding

Training a single model on data from all three paradigms requires a unified labeling scheme with mutually exclusive class assignments. We therefore encoded the four SSVEP, two MI, and two ERP classes as distinct entries in an eight-dimensional one-hot vector, as shown in Figure 2. The four SSVEP classes are assigned to indices 0–3, the left- and right-hand MI classes to indices 4–5, and the ERP target and non-target classes to indices 6–7.

**Figure 2:**
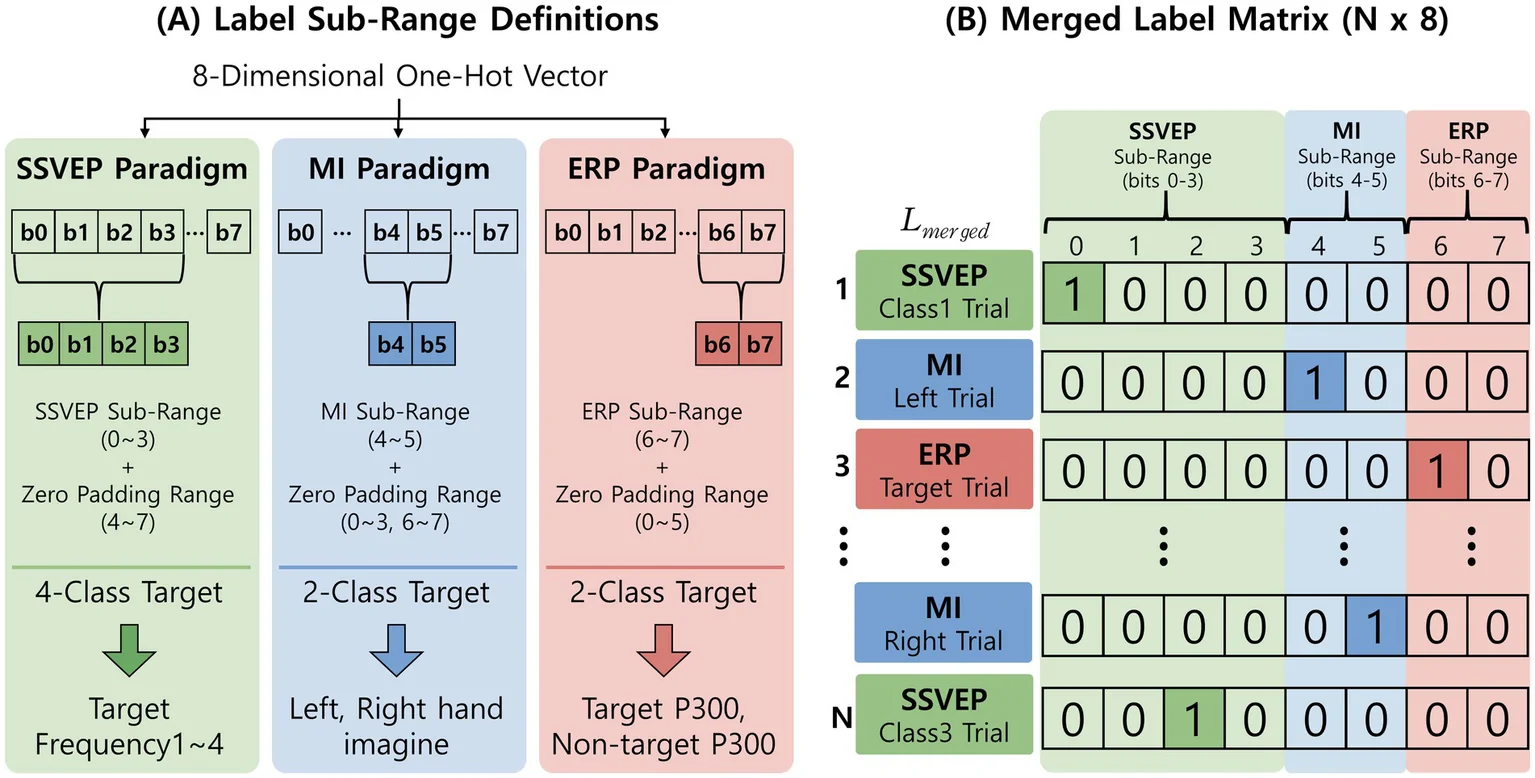
Unified multi-intention label encoding. **(A)** Each trial is represented by an 8-dimensional one-hot vector partitioned into three non-overlapping paradigm sub-ranges: SSVEP (bits 0–3), MI (bits 4–5), and ERP (bits 6–7). All positions outside the active paradigm are set to 0. **(B)** The merged label matrix (*N* × 8) enables joint cross-entropy training across heterogeneous paradigms in a single forward pass.

This encoding merges all three paradigms in the same mini-batch and applies one cross-entropy objective to the eight logits. At inference, the predicted class for each trial is determined by applying argmax over all eight output logits, without paradigm-specific masking. Errors can therefore occur both across and within paradigms, directly testing the shared classifier rather than a paradigm-conditioned subproblem. Under equal class priors, the nominal chance level is 12.5%. Because the observed class counts differ across the eight labels, the grand-average confusion matrix is also reported to reveal class-specific error patterns that are not captured by overall accuracy alone.

### 3.4 Analformer architecture

The Analformer backbone introduced in our earlier study comprises three sequential modules—Analytical Patch Embedding, Transformer Encoder, and Classification Head—as shown in Figure 3. We summarize the reused backbone here for reproducibility. The contribution evaluated in this study is the cross-paradigm extension: a shared eight-class head, joint optimization of MI, ERP, and SSVEP trials, and cohort pre-training followed by participant-specific adaptation.

**Figure 3:**
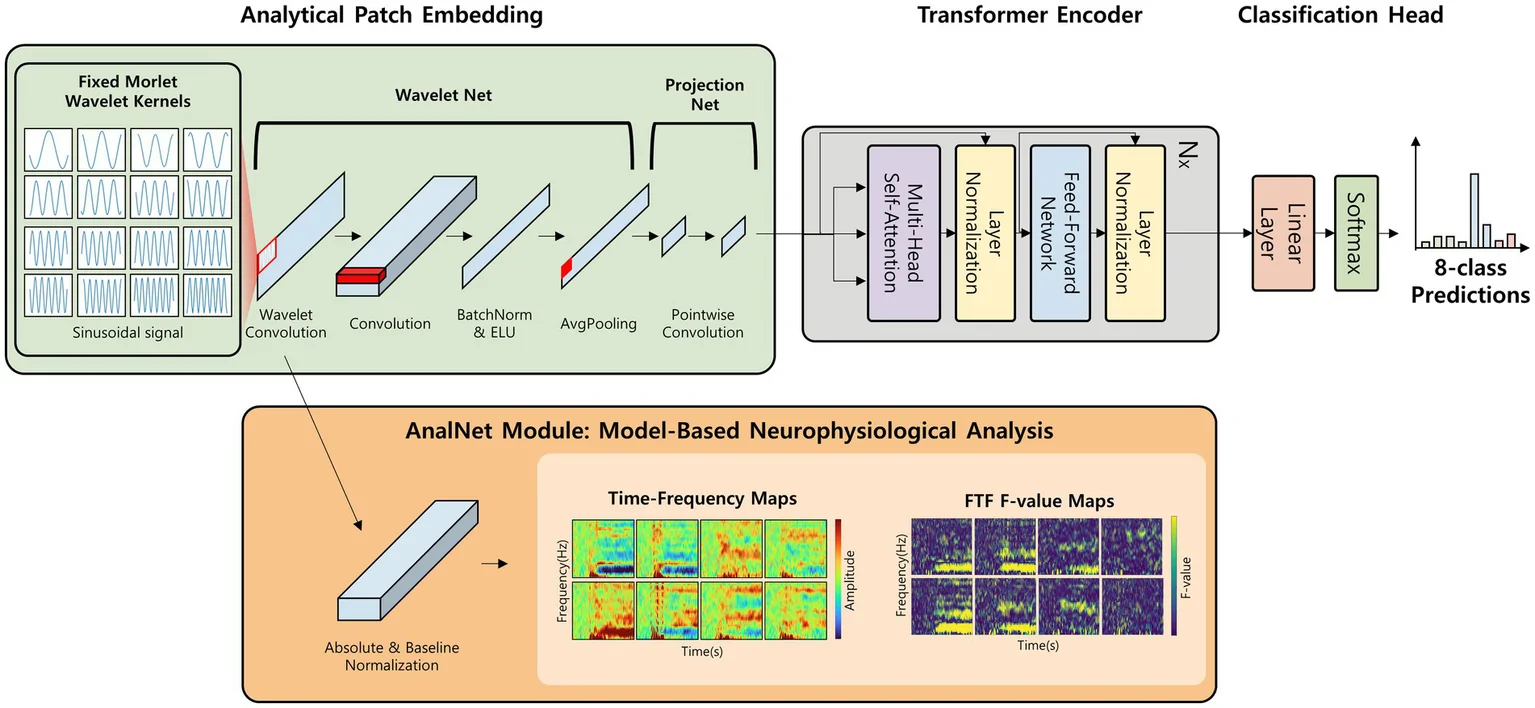
Analformer architecture. Fixed 1–40 Hz Morlet kernels first generate frequency- and channel-resolved activations. A learned convolution combines electrode–frequency responses, average pooling reduces the sequence to 31 positions, and a pointwise projection represents each temporal token with 62 features. Six Transformer blocks integrate these temporal tokens before a multilayer classification head outputs eight logits. AnalNet analyzes the retained fixed-wavelet activations through time–frequency, topographic, and *F*-value time–frequency (FTF) summaries.

#### 3.4.1 Analytical patch embedding

Analytical Patch Embedding transforms each preprocessed EEG epoch into a sequence of temporal tokens for the Transformer. It first applies fixed wavelet-based temporal filtering independently to each electrode and then uses a learned convolution to compress the resulting frequency responses and integrate information across electrodes.

WaveletNet: The first convolution transforms each EEG epoch from an electrode-by-time representation into an electrode-by-frequency-by-time representation. It applies 40 fixed real-valued Morlet kernels, with center frequencies ranging from 1 to 40 Hz in 1-Hz increments, along the temporal axis of each electrode signal. This operation produces 40 frequency-selective temporal responses for each of the 62 electrodes.

The resulting tensor is then rearranged so that the electrodes form the input-channel dimension and the wavelet frequencies form a separate spatial dimension. The second learned convolution uses a 40 × 1 kernel that spans the entire frequency axis. It therefore collapses the 40 frequency responses into a single frequency-compressed representation at each time point. Batch normalization and ELU activation are then applied, followed by average pooling with a 150-sample window and a 10-sample stride, which reduces the temporal sequence to 31 positions. Dropout is subsequently applied for regularization.

AnalNet: AnalNet operates on the output of the first wavelet convolution before the learned frequency-compression layer. It computes the absolute magnitude of each wavelet response and standardizes each frequency–electrode trace using the mean and standard deviation of the prestimulus baseline. These baseline-normalized wavelet responses preserve explicit frequency, electrode, and temporal information and are used to generate time–frequency maps, topographic maps, and *F*-value time–frequency analyses (FTF) without requiring a separate *post hoc* time–frequency decomposition. FTF visualizes the *F*-value of ANOVA depending on frequency over time. The *F*-value is calculated by dividing the variance between groups by the variance within the group (Yeom and Jeong, 2021), as follows:


$$F=\frac{\text{Between}-\text{group variance}}{\text{Within}-\text{group variance}}$$

Further details of the FTF calculation are provided in the original study.

ProjectionNet: Following frequency compression and temporal pooling, a 1 × 1 convolution learns interactions among the 62 feature maps. The resulting tensor is rearranged into 31 temporal tokens, each represented by 62 features. The Transformer input therefore has a shape of 31 × 62, corresponding to 1,922 values when flattened for classification.

#### 3.4.2 Transformer encoder

The Transformer encoder processes the 31 temporal tokens through six pre-normalized encoder blocks. Each block first applies layer normalization followed by 10-head self-attention across the temporal tokens. The attention output is combined with the block input through a residual connection and dropout. A second layer-normalization step is followed by a position-wise feed-forward network with a fourfold expansion, GELU activation, and dropout. Its output is added through a second residual connection, and additional dropout is applied after each encoder block.

#### 3.4.3 Classification head

The classification head flattens the final 31 × 62 representation into a 1,922-dimensional vector and applies a multi-layer perceptron. ELU activation and dropout follow the first two linear layers, and the final layer produces eight logits corresponding to the four SSVEP, two MI, and two ERP classes. Both pooled pre-training and participant-specific adaptation use the standard cross-entropy loss without explicit label smoothing.

### 3.5 Two-stage training strategy

The two-stage protocol first learns a cohort-level representation from OpenBMI Session 1 and then personalizes that representation with the intended user’s Session 1 trials. Table 1 summarizes the settings implemented in Analformer.

**Table 1:** Hyperparameter configuration for the multi-intention experiment.

Hyperparameter	Value
EEG channels (C)	62
Sampling rate (after down sampling)	250 Hz
Epoch window	−0.5 s to +1.5 s (500 samples)
Embedding size/frequency bands (E)	40 (1–40 Hz, 1 Hz steps)
Wavelet kernel width (samples)	50
Avg. pooling window/stride	150/10
Temporal dimension after pooling (Tds)	31
Temporal tokens/feature width	31/62 (flattened size 1,922)
Transformer depth (L)	6
Attention heads (H)	10
Dropout rate	0.2
Batch size	64
Pre-training epochs	500 (L_R = 2 × 10^−4^)
Fine-tuning epochs	500 (L_R = 1 × 10^−4^)
Optimizer/schedule	AdamW; β₁ = 0.9 (pre-training) / 0.5 (fine-tuning), β₂ = 0.999, wd = 5 × 10^−5^; StepLR(200, γ = 0.9)
Output classes	8 (4 SSVEP + 2 MI + 2 ERP)

#### 3.5.1 Pooled session 1 pre-training

The model was optimized on the combined Session 1 data from all three paradigms and all 54 participants. Each participant contributed approximately 600 trials (200 SSVEP, approximately 200 MI, and 200 balanced ERP trials), yielding approximately 32,400 trials with eight-dimensional labels. Pre-training ran for 500 epochs with AdamW (learning rate 2 × 10^−4^, weight decay 5 × 10^−5^) and StepLR decay (step size 200, γ = 0.9). The final pre-training weights initialized participant-specific fine-tuning.

#### 3.5.2 Participant-specific fine-tuning

Following the pre-training phase, the model was adapted to each participant using that participant’s Session 1 data with AdamW (learning rate 1 × 10^−4^, β₁ = 0.5, β₂ = 0.999, weight decay 5 × 10^−5^). Fine-tuning was performed for a fixed budget of 500 epochs.

### 3.6 Evaluation protocol

For each participant, the pretrained model was fine-tuned for 500 epochs using only that participant’s Session 1 data. After the fine-tuning epoch, the current model was evaluated on the corresponding participant’s Session 2 data. The same evaluation procedure was applied to Analformer, the three baseline models, and both ablation models, with each final model evaluated once on the corresponding participant’s Session 2 test data. Session 2 data did not contribute to gradient computation, and the architecture, optimizer, learning-rate schedule, and epoch budget were fixed independently of Session 2 performance. Regarding training, the proposed algorithm underwent 500 epochs of pre-training followed by 500 epochs of fine-tuning, whereas the baseline algorithms were trained for 1,000 epochs.

## 4 Results

### 4.1 Performance evaluation of unified Analformer across three EEG paradigms

Table 2 compares Unified Analformer with EEGNet, ShallowConvNet, and EEG-Conformer after participant-specific adaptation. It reports accuracy for SSVEP, MI, and ERP, along with their unweighted mean.

**Table 2:** Average paradigm-wise and overall classification accuracy (%) across 54 subjects (OpenBMI dataset).

Model	SSVEP (%)	MI (%)	ERP (%)	Mean (%)
Unified Analformer	80.49	66.93	65.59	71.00
EEG-conformer	60.44	61.16	41.98	54.53
EEGNet	48.60	55.55	52.76	52.30
ShallowConvNet	47.21	59.25	29.59	45.35

EEG-Conformer reached 54.53% mean accuracy, EEGNet 52.30%, and ShallowConvNet 45.35% across the three paradigms. Unified Analformer reached 71.00%, exceeding the strongest baseline mean by 16.47 percentage points (Table 2). Because every configuration used the same data partition, training budget, and evaluation procedure, these results provide a protocol-matched comparison within the defined personalized cross-session setting.

Paradigm-specific accuracy was highest for SSVEP at 80.49%, followed by MI at 66.93% and ERP at 65.59%. This ordering is consistent with the fixed frequency-resolved front end matching the narrow-band structure of SSVEP, although the comparison was not designed to attribute between-paradigm differences to a single signal property.

Across 54 participants, Unified Analformer showed the highest mean accuracy for SSVEP, followed by MI and ERP, with substantial between-participant variability in ERP (Figure 4). The grand-average confusion matrix showed 78.2–84.1% diagonal recall across the four SSVEP classes, 65.3 and 68.5% for left- and right-hand MI, and 66.8 and 64.4% for ERP target and non-target trials (Figure 5). The largest individual off-diagonal entries reflected within-paradigm confusions—24.4 and 21.6% between the two MI classes and 16.4 and 16.3% between the two ERP classes—whereas individual cross-paradigm off-diagonal entries were smaller. This error structure supports the ability of the unrestricted eight-class classifier to distinguish classes across paradigms, while highlighting the remaining difficulty of within-paradigm discrimination. These results reflect personalized cross-session decoding, in which pooled Session 1 pre-training and participant-specific adaptation were followed by final-model evaluation on Session 2.

**Figure 4:**
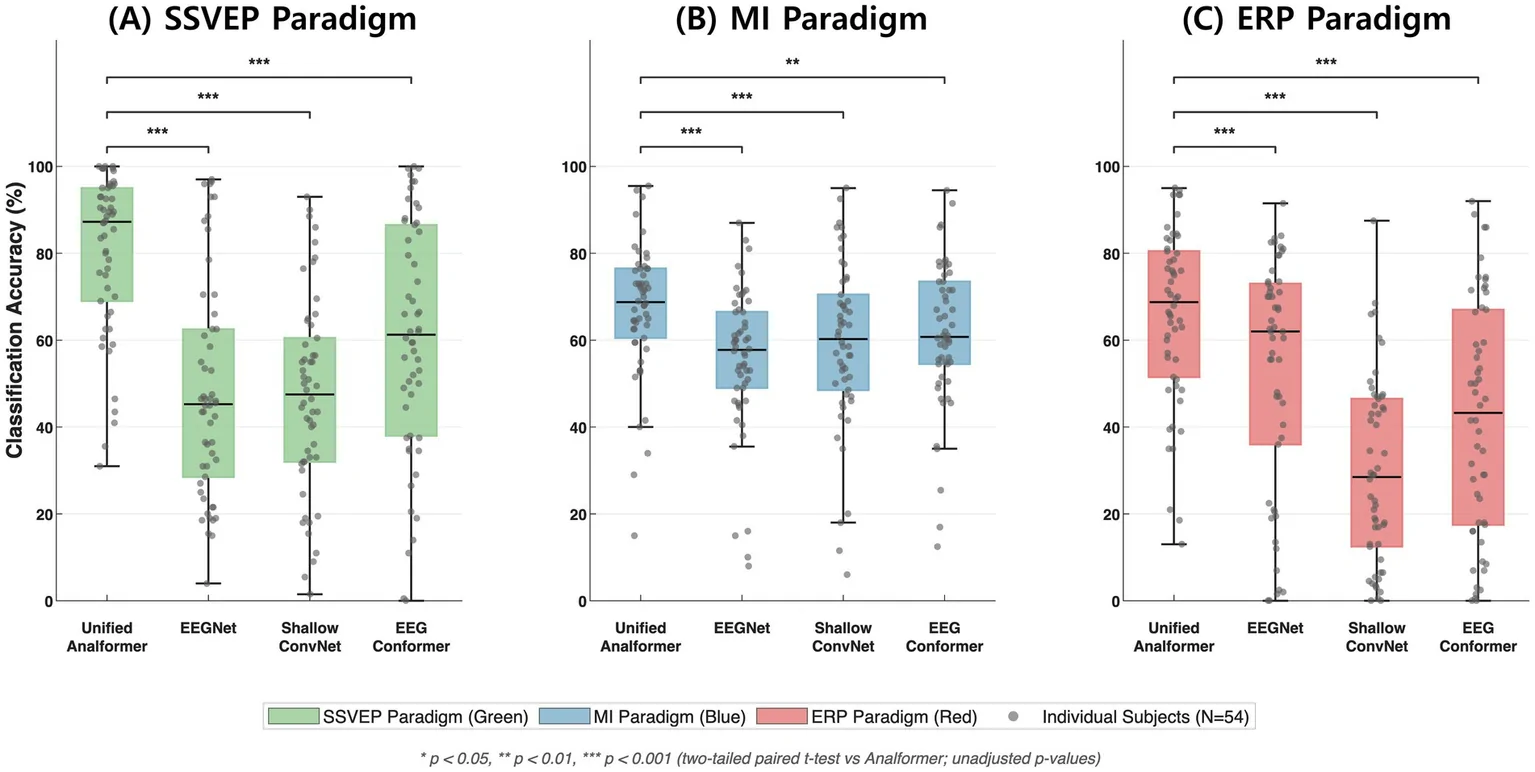
Distribution of per-subject classification accuracy across models for each EEG paradigm. Box plots show the median, interquartile range, outliers, and individual participants (dots) across 54 subjects. **(A)** For SSVEP, the proposed model has the highest observed median and a narrower observed distribution than the baselines. **(B)** For MI, the model distributions overlap more strongly. **(C)** For ERP, all models show greater between-subject variability, and the shallow architectures have lower observed accuracy. Statistical significance was evaluated using two-sided paired *t*-tests between Unified Analformer and each baseline model across the same 54 participants (*p* < 0.05, *p* < 0.01, *p* < 0.001).

**Figure 5:**
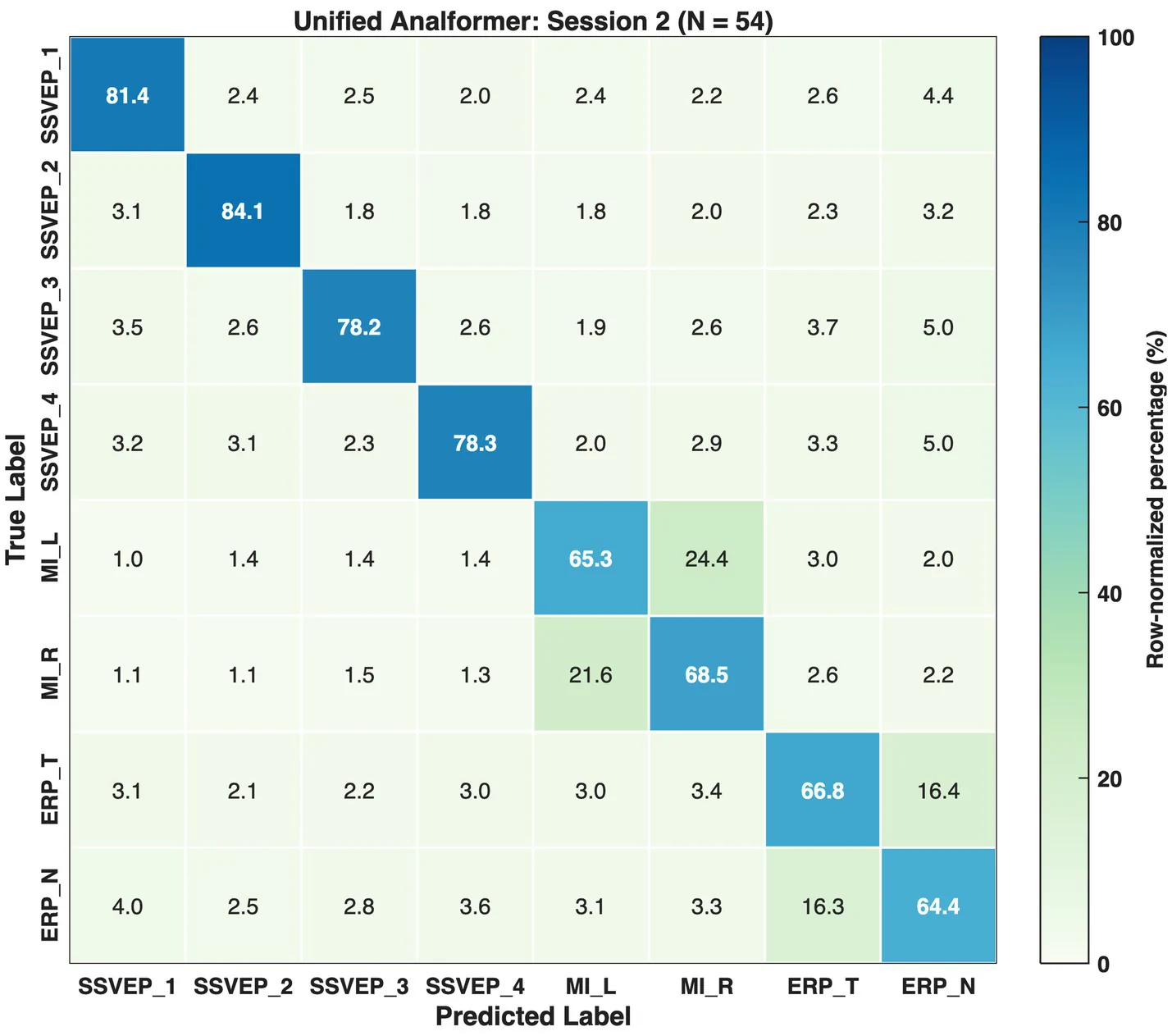
Grand-average confusion matrix across 54 participants. Rows and columns represent the true and predicted labels for SSVEP (four classes), motor imagery (left/right), and ERP (target/non-target). Diagonal concentration indicates class separation in Session 2 after subject-specific adaptation; it does not represent subject-independent generalization.

### 4.2 AnalNet activations recover paradigm-relevant physiological structure

Using Session 1 data, responses from the first fixed wavelet convolution and the parameter-free AnalNet pathway were summarized as *F*-value time–frequency maps without a separate *post hoc* decomposition pipeline. Because neither component contains trainable parameters, these maps are independent of the adaptation epoch and characterize class-discriminative physiological structure present in the input EEG rather than checkpoint-specific learned features. They provide a group-level correspondence check by identifying the channels and frequencies at which activation differences among classes were strongest, without implying sample-level causal attribution.

For MI, the strongest class-discriminative *F*-values occurred over the C3 and C4 sensorimotor regions within the mu (8–13 Hz) and beta (15–30 Hz) ranges (Figure 6a). This anatomical and spectral correspondence shows that the fixed wavelet representation exposes established motor-imagery structure that is available to the subsequent shared decoder.

**Figure 6:**
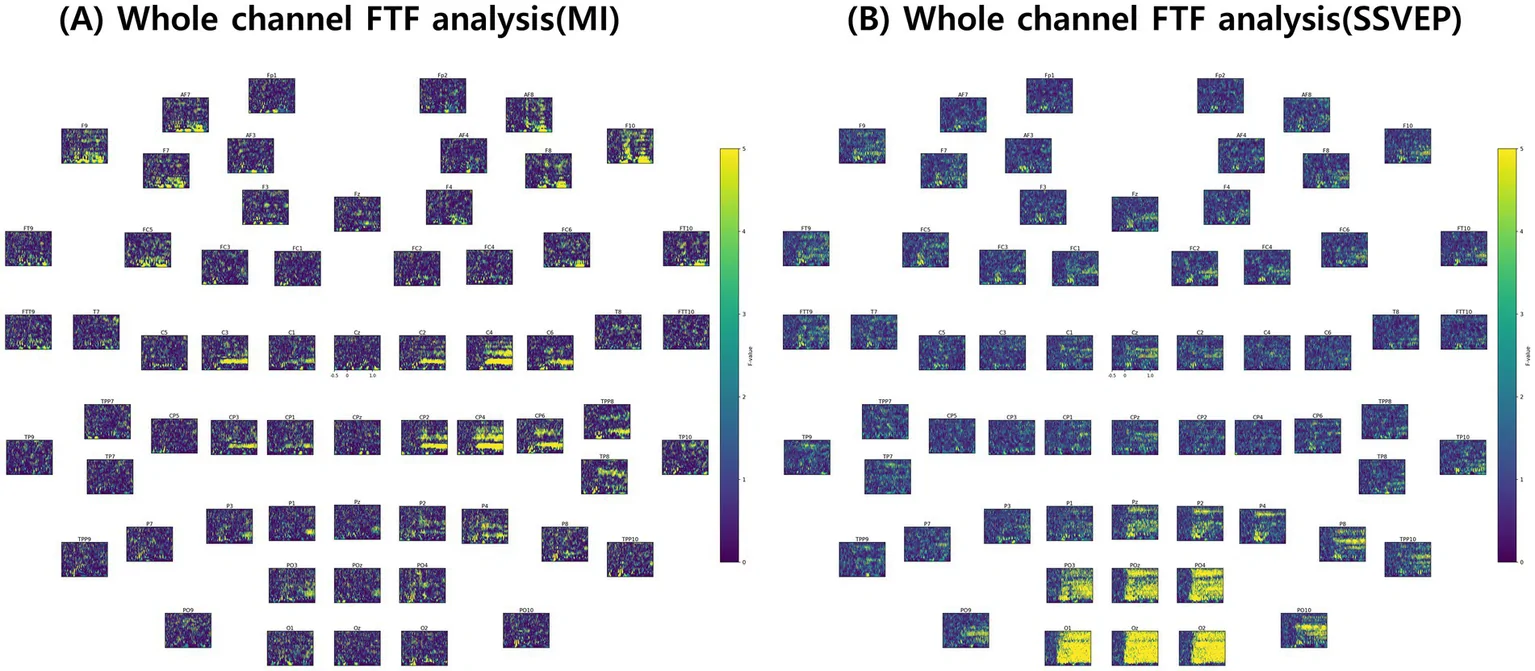
AnalNet-derived *F*-value time-frequency representations. **(a)** MI class discrimination is concentrated over the C3/C4 sensorimotor regions within the mu and beta ranges. **(b)** SSVEP class discrimination is concentrated over O1/O2/Oz at stimulus-related frequencies. The maps summarize group-level representation differences from −0.5 to 1.5 s and do not represent sample-level causal attribution.

For SSVEP, class-discriminative activity concentrated over the occipital channels O1, O2, and Oz at stimulus-related frequencies (Figure 6b). Together, the sensorimotor MI pattern and occipital SSVEP pattern show that the common fixed-wavelet front end exposes complementary structure from both self-initiated and stimulus-driven paradigms. The present AnalNet visualization therefore focuses on MI and SSVEP, whose discriminative signatures are prominently expressed as oscillatory spectral structure. ERP responses are dominated instead by transient low-frequency evoked-potential changes, which are better characterized using latency- and amplitude-sensitive time-domain analyses and were therefore not included in the present wavelet *F*-value visualization.

### 4.3 Component ablations identify contributions from fixed wavelets and pooled pre-training

Table 3 tests the contribution of the fixed Morlet wavelets and the pooled pre-training stage by modifying one component at a time.

**Table 3:** Ablation study.

Configuration	SSVEP (%)	MI (%)	ERP (%)	Mean (%)
Unified Analformer	80.49	66.93	65.59	71.00
Learnable conv. (replace fixed wavelet)	72.65	59.09	57.72	63.15
Fixed wavelet, participant-specific training only	52.10	56.70	33.32	47.38

Each configuration removes or modifies one component relative to the unified Analformer configuration (row 1). Reported as average accuracy (%) across 54 subjects.

Both operational components contributed materially within the evaluated configuration. Replacing fixed Morlet wavelets with learnable convolutions of the same kernel size reduced mean accuracy from 71.00 to 63.15%, with lower values in all three paradigms. Removing pooled pre-training produced the largest observed reduction, from 71.00 to 47.38%, including an ERP decline from 65.59 to 33.32% (Table 3). These ablation results associate the complete model’s observed advantage with both frequency-selective initialization and cohort-level representation learning, with pooled pre-training producing the larger of the two tested contributions.

## 5 Discussion

### 5.1 One shared decision space separates eight classes across three EEG paradigms

Building on the prior paradigm-specific evaluation of Analformer, this study introduces one unrestricted eight-class decision space for MI, ERP, and SSVEP. After pooled Session 1 pre-training and participant-specific adaptation, unified Analformer yielded 71.00% mean Session 2 accuracy and exceeded the means of all three baselines under the same evaluation procedure. The ablations further connect performance to the fixed frequency-selective front end and pooled pre-training. Together, the classification, confusion-matrix, and ablation results show that heterogeneous paradigm-specific EEG responses can remain separable within one participant-adapted architecture.

The principal contribution is the unified label formulation and joint-training protocol, which place four SSVEP, two MI, and two ERP classes under one objective and one output head. By allowing every trial to compete across all eight logits, the unified formulation exposes both within-paradigm and cross-paradigm errors rather than removing them through paradigm-specific masking. The resulting classification and confusion-matrix evidence therefore directly addresses whether one shared head can preserve the distinctions among all eight heterogeneous classes.

### 5.2 Unified decoding provides the classifier core for self-initiated mode switching

Beyond model consolidation, heterogeneous decoding has a concrete control rationale. SMR-based MI and stimulus-driven P300/SSVEP provide complementary functions: MI can be generated without keeping a visual matrix active, whereas P300 and SSVEP can map multiple visual targets to spelling or directional commands. In a future asynchronous hybrid interface, an SMR-derived state could activate an SSVEP direction panel or, as a proposed extension, a P300 speller; the visual paradigm could then provide the larger command set, and another SMR-derived state could suspend stimulation when the user returns to idle. This hierarchy could make typing or movement available on demand while limiting unnecessary flashing or flickering during non-control periods. This division of roles could preserve the command richness of stimulus-driven interfaces while reducing unnecessary visual-stimulation exposure and enabling user-initiated access (Pfurtscheller et al., 2010).

The present experiment establishes the offline feasibility of the shared classifier layer by separating eight labels across three cue-paced paradigms after participant-specific adaptation. A closed-loop implementation can add an idle/no-control detector around this core, allowing SMR-derived states to govern when the P300 or SSVEP interface is active. In that system-level extension, false activation rate, transition latency, command-selection accuracy, visual-stimulation exposure, and user workload become direct measures of on-demand hybrid control.

AnalNet revealed class-discriminative spatial-frequency patterns corresponding to sensorimotor mu/beta activity over C3 and C4 for MI and frequency-locked occipital responses over O1, O2, and Oz for SSVEP. These findings show that the fixed-wavelet representations retain spatial-frequency patterns consistent with established neurophysiological characteristics of MI and SSVEP.

AnalNet is used here to test representation-level physiological coherence, not to claim sample-level causal attribution. That scope is sufficient for the present contribution: accuracy establishes separability, whereas the AnalNet maps test whether the retained representations remain aligned with established EEG physiology. Feature perturbation can extend this representation-level evidence to decision-level attribution without changing the conclusion that one shared classifier preserves distinct MI and SSVEP structure.

### 5.3 Ablations link performance to frequency structure and pooled pre-training

The wavelet ablation is consistent with fixed 1-Hz Morlet filters supplying a useful cross-paradigm inductive bias. The filters directly expose SSVEP stimulation frequencies, MI band-power changes, and low-frequency ERP content before learned mixing, while the Transformer integrates the resulting temporal sequence.

Recent studies have explored adaptive and learnable wavelet formulations that allow time–frequency representations to be adjusted directly from the data. In this study, we retained fixed Morlet wavelets to provide a common 1–40 Hz frequency basis across MI, ERP, and SSVEP and to support direct physiological interpretation. This fixed frequency basis allows paradigm-specific spectral patterns to be examined within a common frequency range. These results do not suggest that fixed kernels are generally superior to learnable wavelets. Rather, they show that, within the proposed Analformer framework and experimental setting, fixed Morlet wavelets provide a useful and interpretable representation compared with the standard learnable convolution used in the ablation.

The larger reduction without pooled pre-training identifies cohort-level representation learning as the dominant tested contributor before participant-specific adaptation. Because each ablation changes one operational component while using the same procedure, the contrasts quantify how much the complete configuration depends on frequency-selective initialization and pooled learning.

### 5.4 A fixed-horizon adaptation protocol enables protocol-matched comparison

This study employs a two-stage training procedure for Analformer, consisting of 500 epochs of pooled pre-training followed by 500 epochs of participant-specific fine-tuning.

After completion of the predefined training procedure, the final model was evaluated once on the test data. Test performance was not used for checkpoint selection, early stopping, hyperparameter tuning, or any other model-selection decision. The same preprocessing and final-model evaluation protocol was applied to all compared models and ablation settings.

Under this evaluation procedure, unified Analformer maintained the highest mean across SSVEP, MI, and ERP and exceeded the strongest baseline mean by 16.47 percentage points. The ablations provide convergent contrasts: replacing fixed wavelets lowered mean accuracy by 7.85 points, whereas removing pooled pre-training lowered it by 23.62 points. These matched differences link the observed advantage to the framework’s two defining components.

The experimental scope represents personalized cross-session deployment on OpenBMI: learn a cohort-level representation, adapt it with the intended user’s Session 1 data, and evaluate the personalized decoder in a later session. This setting matches BCI operation in which user-specific calibration precedes subsequent control. Likewise, the cue-paced, mutually exclusive trials isolate the shared eight-class decision layer, allowing asynchronous state detection and temporal control logic to be added as dedicated system modules around a validated classifier core.

## 6 Conclusion

We extended Analformer into one unrestricted decoder for four SSVEP, two MI, and two ERP classes. Fixed Morlet kernels preserve frequency-resolved EEG structure, learned electrode–frequency mixing produces 31 temporal tokens, and six Transformer blocks integrate those tokens before one eight-class head assigns each trial across all three paradigms.

With all eight classes competing in one unrestricted output space, the nominal chance level under equal class priors was 12.5%. Analformer reached 71.00% mean accuracy and exceeded the strongest baseline by 16.47 percentage points under the same evaluation conditions. Accuracy decreased to 63.15% when learnable convolutions replaced the fixed wavelets and to 47.38% without pooled pre-training. AnalNet further localized MI discrimination to sensorimotor mu/beta regions and SSVEP discrimination to stimulus-related occipital activity, providing physiological evidence that the shared objective retained paradigm-specific structure.

Together, the unrestricted classification, protocol-matched ablations, and physiological correspondence results support the feasibility of participant-adapted unified decoding as a shared classifier core for hybrid BCIs. Adding idle-state detection and temporal control logic around this core can support on-demand P300/SSVEP selection while limiting visual stimulation to periods of intended use.

## Data Availability

Publicly available datasets were analyzed in this study. This data can be found at: https://gigadb.org/dataset/100542.
